# Retraction: Wang et al. Cardiovascular Disease and Exercise: From Molecular Mechanisms to Clinical Applications. *J. Clin. Med.* 2022, *11*, 7511

**DOI:** 10.3390/jcm12062143

**Published:** 2023-03-09

**Authors:** Bo Wang, Lin Gan, Yuzhi Deng, Shuoji Zhu, Ge Li, Moussa Ide Nasser, Nanbo Liu, Ping Zhu

**Affiliations:** 1 Guangdong Cardiovascular Institute, Guangdong Provincial People’s Hospital, Guangdong Academy of Medical Sciences, Guangzhou 510100, China; xnwangbo430@hotmail.com (B.W.);; 2 Guangdong Provincial Key Laboratory of Pathogenesis, Targeted Prevention and Treatment of Heart Disease, Guangzhou 510640, China

The *Journal of Clinical Medicine* retracts the article entitled “Cardiovascular Disease and Exercise: From Molecular Mechanisms to Clinical Applications” [1].

Following publication, concerns were brought to the attention of the publisher regarding overlap with a previously published manuscript [2] with a different authorship group.

Adhering to our complaint’s procedure, an investigation was conducted that confirmed the extensive use and dispersed nature of the overlap and the article is therefore retracted. 

This retraction was approved by the Editor in Chief of the *Journal of Clinical Medicine*. 

The authors agreed to this retraction.
